# High Di-rhamnolipid Production Using *Pseudomonas aeruginosa* KT1115, Separation of Mono/Di-rhamnolipids, and Evaluation of Their Properties

**DOI:** 10.3389/fbioe.2019.00245

**Published:** 2019-10-22

**Authors:** Jie Zhou, Rui Xue, Shixun Liu, Ning Xu, Fengxue Xin, Wenming Zhang, Min Jiang, Weiliang Dong

**Affiliations:** ^1^State Key Laboratory of Materials-Oriented Chemical Engineering, College of Biotechnology and Pharmaceutical Engineering, Nanjing Tech University, Nanjing, China; ^2^Jiangsu National Synergetic Innovation Center for Advanced Materials (SICAM), Nanjing Tech University, Nanjing, China

**Keywords:** high-yielding, di-rhamnolipids, separation, CMC, emulsifying activity

## Abstract

Rhamnolipids (RLs) are important bioproducts that are regarded as promising biosurfactant for applications in oil exploitation, cosmetics, and food industry. In this study, the newly isolated *Pseudomonas aeruginosa* KT1115 showed high production of di-RLs. The highest yield of RLs by *P. aeruginosa* KT1115, reaching 44.39 g/L after 8 days of fermentation in a 5 L bioreactor, was obtained from rapeseed oil-nitrate medium after process optimization. Furthermore, we established a new separation process that achieved up to 91.82% RLs recovery with a purity of 89% and further obtained mono/di-rhamnolipids. Finally, ESI-MS analysis showed that the RLs produced by strain KT1115 have a high proportion of di-RLs (mono-RLs: di-RLs = 11.47: 88.53), which have a lower critical micelle-forming concentration (8 mN/m) and better emulsification ability with kerosene (52.1% EI_24_) than mono-RLs (167 mN/m and 41.4% EI_24_, respectively). These results demonstrated that *P. aeruginosa* KT1115 is a potential industrial producer of di-RLs, which have improved applicability and offer significant commercial benefits.

## Introduction

Rhamnolipids (RLs), which are mainly produced by *Pseudomonas aeruginosa*, are the most intensively studied glycolipid biosurfactants (Seul Ki et al., 2011; Sodagari et al., 2013). Rhamnolipid surfactants were shown to reduce the surface tension of water from 72 to 28 mN/m, and the interfacial tension of water-oil systems from 43 to <1 mN/m (Liang-Ming et al., 2008). RLs have significant advantages in emulsification, wetting, dispersion, dissolution, and decontamination. Consequently, they have been widely used in oil exploration, cosmetics, food, and other industries owing to their high specificity, biodegradability, and biocompatibility (Mulligan, 2005).

RLs are synthesized by covalently linking one or two rhamnose moieties with one or two hydroxy fatty acids, and the corresponding types are designated as mono- or di-RLs based on the number of rhamnose units (Rendell et al., 1990). The components and properties of RLs are profoundly influenced by the producing strains and cultivation conditions (Rahman et al., 2010). For instance, the ratio of mono- and di-RLs produced by *P. aeruginosa* mainly lies between 40:60 and 30:70 (Matasandoval et al., 1999; Jadhav et al., 2018). The percentage of mono-RLs even reaches 68.35% of the total RL content produced by *P. aeruginosa* MN1 (Arino et al., 1996; Samadi et al., 2012). Compared to mono-RLs, di-RLs possess higher surface activity, and therefore show great potential in oily sludge dewatering and lignocellulose degradation (Xuwei et al., 2013; Jiang et al., 2014). However, there is little available information in terms of systematic comparative analysis of the physicochemical properties of mono- and di-RLs.

To better understand the characteristics of different RLs components, separation, and purification are the first and most important step. More than half of the cost of RLs production is due to downstream processing, and is still no economical and reliable downstream technology for the recovery and purification of RLs at the industrial scale (Roger and Banat, 2012; Rienzo et al., 2016). The most commonly employed separation techniques for the isolation of RLs from the fermentation broth include foam separation, membrane separation and adsorption (Isa et al., 2007; Rienzo et al., 2016; Jadhav et al., 2018). However, these methods are mainly used for crude RLs separation, and the lack of a quick and effective separation method is the main bottleneck holding back research on mono- and di-RLs.

In our previous study, a RLs-producing strain was successfully isolated from oil-contaminated soil, and was identified as *P. aeruginosa* KT1115. To achieve high RLs production using *P. aeruginosa* KT1115, process optimization in a rapeseed oil-nitrate medium was first conducted. Afterwards, a rapid and efficient purification and separation method for mono- and di-RLs was established. Finally, a comprehensive physicochemical characterization of the isolated mono- and di-RLs was carried out.

## Materials and Methods

### Strains, Media, and Culture Conditions

*Pseudomonas aeruginosa* KT1115 was isolated from soil samples using CTAB screening (Varjani and Upasani, 2016). Luria-Bertani (LB) medium containing 10 g/L tryptone, 5 g/L yeast extract and 10 g/L NaCl was used for seed culture. The rapeseed oil-nitrate medium (RON) containing 60 g/L rapeseed oil (Jinlongyu, China), 6 g/L NaNO_3_, 3 g/L yeast extract, 1 g/L KH_2_PO_4_, 1 g/L Na_2_HPO_4_, 0.1 g/L CaCl_2_·2H_2_O, and 0.1 g/L MgSO_4_ was used for fermentation.

All chemicals and solvents were of analytical grade and purchased from Sahn Chemical Technology Co. Ltd. (Shanghai, China). Aliquots comprising 100 mL seed and fermentation medium were dispensed into Erlenmeyer flasks (500 mL) and sterilized at 121°C for 20 min. Seed culture and bacterial fermentation were performed at 37°C and 180 rpm for 8 days. All experiments were performed in triplicates.

### Single-Factor Optimization

Single factor experiments were applied to optimize the liquid fermentation conditions. The influence of different pH values (5.5, 6, 6.5, 7, 7.5, 8, and 9) concentrations of metals including calcium (0, 0.5, 1, 5, and 10 mM) and magnesium (0, 0.5, 1, 2, 5, and 10 mM), rapeseed oil concentrations (10, 20, 40, 60, 80, and 100 g/L), yeast extract concentrations (0, 3, 6, 9, and 12 g/L), NaNO_3_ concentrations (0, 3, 6, 9, and 12 g/L), KH_2_PO_4_ concentrations (0, 1, 2, 5, 10, 20, and 50 g/L), Na_2_HPO_4_ concentrations (0, 1, 2, 5, 10, 20, and 50 g/L), and different inoculum sizes (1, 5, 10, 15, and 20%) on the production of RLs was evaluated. Quantitative analysis of rhamnolipids was conducted using anthrone colorimetry (Heyd et al., 2008).

### Extraction and Purification of Rhamnolipids

RLs products were extracted based on a procedure described previously (Déziel et al., 1999), with minor modifications as follows: The bacterial cells were first removed by centrifugation (15,000 × g, 20 min), after which different concentrations of ethanol (10, 20, 30, 40, and 50%) were added to the supernatant to precipitate proteins. The samples were kept at 4°C for different times (3, 6, 9, and 18 h). The supernatants were centrifuged at 15,000 × g for 10 min, and the pH value was adjusted to 2.0 using different acids (H_3_PO_4_, H_4_SO_4_, HCl, and HNO_3_). Then, the acidified supernatants were kept at 4°C for different times (3, 6, 9, and 18 h) to precipitate the RLs. The precipitates were centrifuged 15,000 × g for 10 min and the crude RLs were placed into a drying oven at 90°C for 10 h. After that, petroleum ether was added to the anhydrous precipitated RLs for purification and centrifugation (15,000 × g for 10 min) to obtain the purified rhamnolipids, which were used for further analysis.

### ESI-MS Analysis

The RLs were dissolved in methanol solution, and RLs solutions with a concentration of 1 mg/mL were prepared for congener composition analysis by electrospray ionization mass spectrometry (ESI-MS). The ESI mass spectrum in negative ion mode was acquired using a capillary voltage of −3.5 kV, fragmentation voltage of 50 V and nitrogen as desolvation gas, which was heated to 325°C, at flow rate of 7 L/min. ESI tandem mass spectra were acquired by mass-selecting the target ion using the quadrupole mass analyzer followed by 20 eV collision-induced dissociation using nitrogen in the collision cell. The mass spectrometer was operated in negative ion mode scanning in the 50–1,000 m/z range.

### Separation of Mono- and Di-rhamnolipids

Silica gel column chromatography was used to further separate mono-/di-RLs. The sample was loaded on top of the silica gel column and eluted with five different chloroform-methanol solvent systems of gradually increasing polarity. The five mobile phases CHCl_3_:CH_3_OH (v/v) were used in sequence: solution I was chloroform (100%), which was primarily used to remove the neutral lipids and other impurities, and solution II, solution III was chloroform/methanol (v/v) 97:3, which was primarily used to elute the mono-RLs and solution IV, solution V (chloroform/methanol = 93:7) to elute the di-RLs. Finally, the separation result was visualized by thin layer chromatography (TLC) using chloroform/methanol/H_2_O (65:7:2) as mobile phase (Samadi et al., 2012).

### Determination of Critical Micelle Concentrations

Aqueous solutions of the RLs at different concentrations solutions (0–1,000 mg/L) were prepared to determine the critical micelle concentration (CMC) using the break point of the surface tension vs. the log of the bulk concentration curve. For the calibration of the instrument, the surface tension of pure water was measured before each set of experiments. These experiments were conducted in triplicates and the results were presented as averages.

### Analysis of Emulsifying Activity

Solutions of the extracted RLs and purified mono/di-RLs at a concentration of 100 mg/L were prepared to determine the emulsification index, and compared with SDS and Tween 80 under the same conditions. A hydrophobic phase comprising 4 mL of hydrophobic organics (kerosene, liquid paraffin, and n-hexane, respectively) was mixed with 4 mL of the RLs solutions in the test tubes. The tubes were stirred using a vortex mixer for 2 min and the height of the emulsion layer was measured after 24 h to determine the emulsification index (EI_24_(%); Lovaglio et al., 2011).

### Evaluation of the Stability of the Extracted RLs and Purified Mono/Di-RLs

The EI_24_ index were measured using liquid paraffin as emulsion phase to evaluate the stability of the extracted RLs and purified mono/di-RLs at a concentration of 100 mg/L. H_3_PO_4_ and NaOH at a concentration of 1 M were used to adjust the pH values. A series of solutions comprising 20 mL of RLs fraction were treated at different temperatures (4, 10, 20, 30, 40, 50, 60, 70, 80, and 90°C), different pH values (2, 4, 6, 8, 10, and 12), and different salinities (NaCl concentrations of 0, 1, 2, 4, 6, 8, 10, and 12%).

## Results and Discussion

### Fermentation Optimization

**Figure 1:**
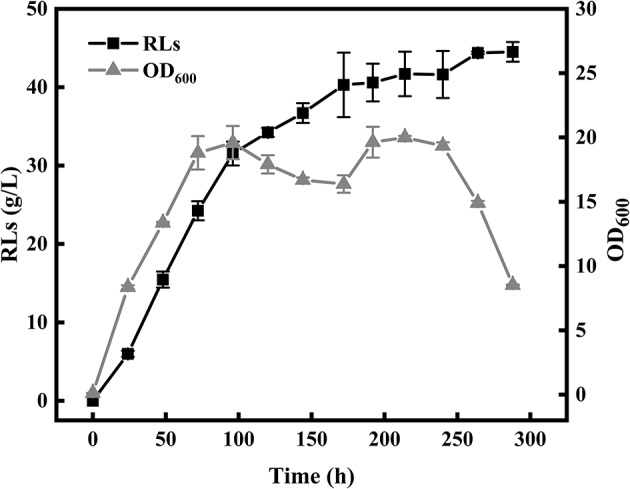
Bioreactor fermentation for rhamnolipid production.

The color change of the anthrone reagent is positively associated with the rhamnolipid concentration (100–800 mg/L). The linear correlation model between the diameters of oil spreading circle (y) and rhamnolipid concentrations (x) was described by an empirical equation as follows: y = 330.21 × −22.34 (*R*^2^ = 0.9979). According to the color change of the anthrone reagent, the amount of RLs in the culture was calculated using two positive linear correlation models. The single factor experiments showed that the medium composition and optimal fermentation conditions leading to the highest RLs production encompassed 60 g/L rapeseed oil, 6 g/L NaNO_3_, 3 g/L yeast extract, 1 g/L KH_2_PO_4_, 1 g/L Na_2_HPO_4_, 0.1 g/L CaCl_2_·2H_2_O, 0.1 g/L MgSO_4_, and inoculation at 1% inoculum size, followed by cultivation at 37°C, pH 7.5 and 180 rpm for 8 days. The strain KT1115 produced 44.39 g/L RLs after 10 days of fermentation in a 5-L bioreactor under the optimal fermentation conditions as shown in Figure 1.

### Extraction and Purification of the Produced RLs

Because the extraction and purification of RLs is key to any detailed study of their characteristics, we established a new separation protocol consisting of four steps including cell removal, protein removal, acid precipitation, and petroleum ether purification.

**Figure 2:**
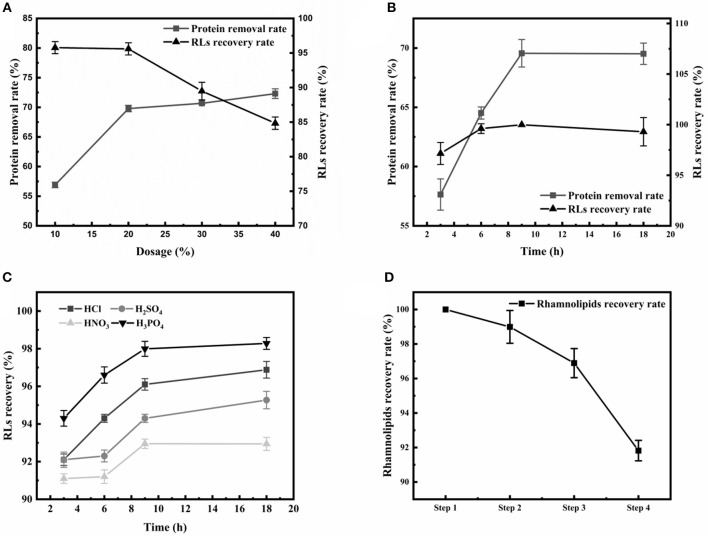
Optimization of the crude RLs separation process. **(A)** The effects of different ethanol concentrations on the rhamnolipid recovery rate and protein removal rate; **(B)** The effects of different durations of ethanol precipitation on the rhamnolipid recovery rate and protein removal rate; **(C)** The effects of different acids on the RLs recovery rate. **(D)** Summary of purification of rhamnolipids from the fermentation broth. The rhamnolipid sample concentration was 10 g/L. The rhamnolipid recovery rate is calculated by comparing the pre- and post-treatment concentrations.

Firstly, the appearance of emulsions is a great challenge during the extraction of RLs. Large amounts of proteins were present in the RLs fermentation broth, and were identified as an essential cause of emulsification (Damodaran, 2010). Thus, after removing the cells by centrifugation, an ethanol precipitation step for eliminating as much protein as possible from the broth was applied before RLs extraction. The effects of various ethanol concentrations and treatment times on protein removal rate and RLs recovery rate were determined (Figures 2A,B). The protein removal rate gradually increased with the increase of the ethanol concentration, and the increase of the protein removal rate and RLs recovery rate slowed down at concentrations exceeding 20% (Figure 2A). The optimal treatment time was 9 h as shown in Figure 2B.

After removing the proteins, acid precipitation was used to further extract RLs, and the effects of different acids on RLs precipitation were investigated (Figure 2C). The results showed that a higher RLs recovery rate was obtained by using H_3_PO_4_ as the acid precipitating reagent at pH values of 2.0. Finally, petroleum ether was used to further purify the rhamnolipids, which are insoluble in petroleum ether, so that residual fats and lipid-soluble pigments in the rhamnolipid solution could be removed. Using this newly established four-step protocol, the final RLs product was separated from the fermentation broth with a high recovery rate of 91.82% and good purity of 89% (Figure 2D).

### Analysis of the Molecular Composition of the Produced Rhamnolipids

**Figure 3:**
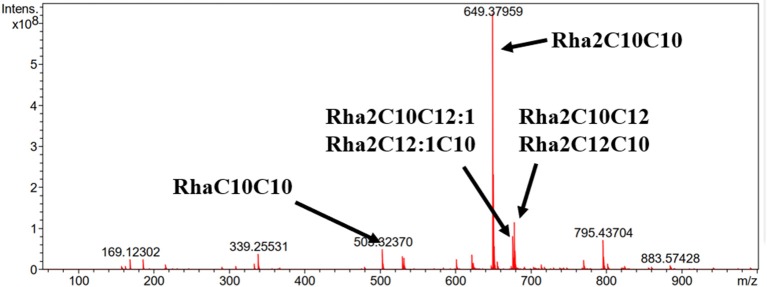
ESI-MS analysis of the produced rhamnolipids.

ESI-MS was used to analyze the molecular composition of the RLs separated by acid precipitation as shown in Figure 3. The results revealed the presence of many RL homologs produced by *P. aeruginosa* KT1115 during growth on RON medium, with a major ion peak at m/z 649. As shown in Table 1, the RLs products encompassed both mono- and di-RLs, but di-RLs such as Rha_2_C_10_C_10_ at m/z 649 were detected as the major components. The results shown in Table 2 indicate that the KT1115 strain has excellent di-RLs production ability compared to previous reports. The four predominant RLs homologs were Rha_2_C_10_C_10_, Rha_2_C_10_C_12_, Rha_2_C_10_C_12:1_, and RhaC_10_C_10_ at 63.37, 10.59, 7.40, and 4.41% of the total, respectively. In addition, all di-rhamnolipid congeners added up to 88.5% of the total congeners in the biosurfactant mixture. This result demonstrates that *P. aeruginosa* KT1115 is a high di-rhamnolipid-yielding strain.

**Table 1:** Structural composition of rhamnolipids produced by strain KT1115.

**Homologue**	**Pseudomolecule ion (m/z)**	Ratio (%)^*^
Rha_2_C_10_C_10_	649	65.37
Rha_2_C_10_C_12_	677	10.59
Rha_2_C_12_C_10_		
Rha_2_C_10_C_12:1_	675	7.40
Rha_2_C_12:1_C_10_		
RhaC_10_C_10_	503	4.41

* *The homologues with abundances of less than 4.41% are not listed*.

**Table 2:** Comparison of mono/di-rhamnolipids from different RL-producing strains.

**Strain**	**Carbon source**	**Mono-/di-RLs**	**References**
*P. aeruginosa* UG2	Corn oil	28:72	Matasandoval et al., 1999
*P. aeruginosa* MTCC 2453	Sunflower oil	25.5:74.5	Jadhav et al., 2018
*P. aeruginosa* 47T2	Waste fried oil	21:79	Sánchez et al., 2007
*P. aeruginosa* MN1	Glycerol	76.48:23.52	Samadi et al., 2012
*P. aeruginosa* 57RP	Mannitol	31.75:68.25	Déziel et al., 1999
*P. aeruginosa* 57RP	Naphthalene	38.90:61.10	Déziel et al., 1999
*P. aeruginosa* GL1	Glycerol	69:31	Arino et al., 1996
*P. aeruginosa* ADMT1	Diesel	33:67	Tiwary and Dubey, 2018
*P. aeruginosa* MR01	Soybean oil	50.73:49.27	Lotfabad et al., 2010
***P. aeruginosa*** **KT1115**	**Rapeseed oil**	**11.47:88.53**	**This work**

*Bold values indicates the rhamnolipid mixture, the component of the di-rhamnolipid accounted for 88.53%*.

### Separation of Mono- and Di-rhamnolipids

**Figure 4:**
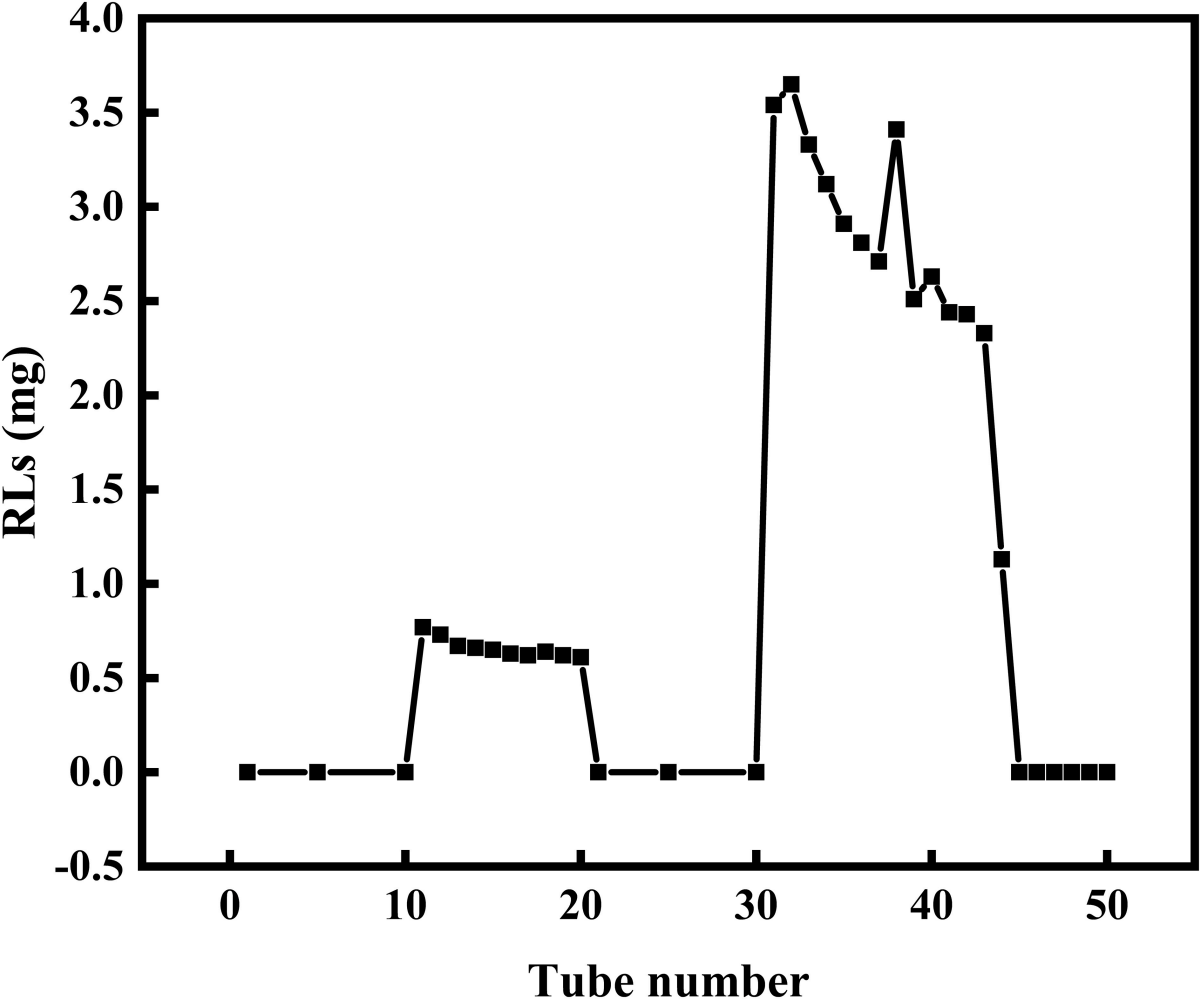
The separation of mono- and di-RLs by silica gel column chromatography. Mono- and di-RLs were separated by gradient solvent elution. Tubes 0–10 were eluted with solvent II. Tubes 11–20 were eluted with solvent III to release mono-RLs. Tubes 21–30 tube were eluted with solvent IV, which confirmed that mono-RLs were entirely eluted because nothing was eluted in this fraction. Tubes 31–40 were eluted with solvent V to release di-RLs from the column.

**Figure 5:**
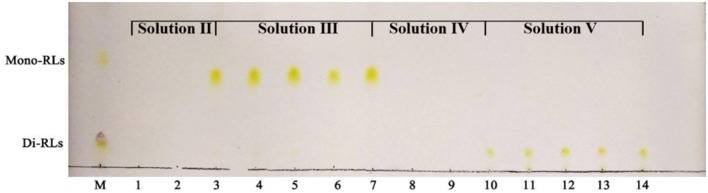
TLC analysis of mono- and di-rhamnolipids separated by silica gel column chromatography. M, commercial rhamnolipids; Lanes 1,2, the samples eluted by solvent II; Lanes 3–7, the samples eluted by solvent III; Lanes 8,9, the samples eluted by solvent IV; Lanes 10–14, the samples eluted by solvent V.

Silica gel column chromatography was used to separate mono- and di-RLs utilizing the polarity difference between the two RLs types. The eluent solution was assessed for RLs concentration as shown in Figure 4. As the polarity of the eluent increased, mono- and di-RLs were eluted successively. The results of thin-layer chromatography (TLC) suggested that the mono-/di-RLs produced by *P. aeruginosa* KT1115 were completely separated. In the TLC chromatogram, the lower spots are di-rhamnolipid structures, and the higher spots contain mono-rhamnolipid molecules (Figure 5).

### Evaluation of the Surface-Active Properties of the Purified Mono- and Di-RLs

#### Critical Micelle Concentrations

**Figure 6:**
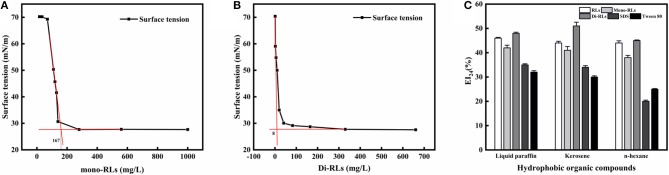
The evaluation of surface tension and emulsifying activity. Relationship between the surface tension and concentration of mono-RLs **(A)** and di-RLs **(B)**; Emulsifying activity of five surfactants was assessed using three hydrophobic organic compounds **(C)**. The EI_24_ of the five surfactants at 100 mg/L were measured and their emulsifying activities with different substrates (kerosene, liquid paraffin, n-hexane)0020were compared.

The critical micelle concentration (CMC) is the concentration of a surfactant in a bulk phase, above which aggregates of surfactant molecules, so-called micelles, start to form. It is an important parameter for assessing the interfacial activity of surfactants (Elshikh et al., 2017). As the concentration of RLs increased, the surface tension value rapidly decreased, resulting in a sharp turn as shown in Figures 6A,B. And the surface tension dose not drop further above the CMC, which is extensively independent of the RLs concentration. The CMC can be calculated using the intersection between the regression straight line of the linearly dependent region and the straight line passing through the plateau. As determined by surface tension measurements, the CMC value of purified mono-RLs was 167 mN/m, whereas the value of purified di-RLs was much lower at 8 mN/m, proving that di-RLs have a better surface activity and are better biosurfactants than mono-RLs, as had been conjectured for a long time based on their respective structures. To the best of our knowledge, this is the first time that the CMC values of purified mono- and di-RLs were exactly determined.

#### Emulsifying Activity

The emulsifying activity of five surfactants, including RLs, mono-RLs, di-RLs, SDS, and Tween 80, was assessed using three hydrophobic organic compounds, namely, liquid paraffin, kerosene, and n-hexane. All RLs products showed a higher emulsifying activity than either SDS or Tween 80, and the EI_24_ values of RLs, mono-RLs and di-RLs with the three tested hydrophobic organic compounds were all >40% (Figure 6C). The high emulsifying activity of rhamnolipids makes them suitable for bioremediation of oil-polluted sites and application in microbial enhanced oil recovery (MEOR) (Wen-Jie et al., 2012).

#### Effects of Temperature, pH, and Ionic Strength on Biosurfactant Stability

**Figure 7:**
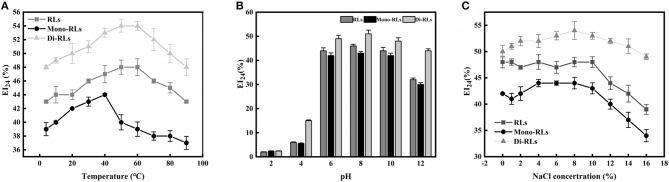
The stability of rhamnolipids. **(A)** Thermostability; **(B)** pH stability; **(C)** salt stability.

The temperature and pH profiles of the emulsifying activity of the RLs products are shown in Figures 7A,B. The results indicated that temperature had little effect on the biosurfactant performance in a broad range from 20 to 80°C, whereby di-RLs had the best emulsification capacity. However, the emulsifying activity of RLs was significantly affected by the pH (Figure 7B). The emulsification activity of RLs was weak under strongly acidic conditions (pH 2–4). Nevertheless, the EI_24_ value of purified di-RLs was relatively stable under a wide range of pH values (4.0–12.0).

The effect of ionic strength on the emulsification capacity of different RLs products is shown in Figure 7C. The EI_24_ value was strongly reduced as the ionic strength was increased up to 10% NaCl concentration. As suggested above, purified di-RLs showed a better stability than purified mono-RLs.

## Discussion

Rhamnolipids (RLs), which are mainly produced by *P. aeruginosa*, have attracted the attention of researcher as biosurfactants with many potential industrial applications. There are many reports underscoring the superior biosurfactant properties of RLs, with promising potential applications such as biofilm disrupting, skin damage repair and perhaps even anticancer activity (Stipcevic et al., 2006; Kamal et al., 2012; Nivedita et al., 2013). However, the RLs secreted by *P. aeruginosa*, which has been used to obtain most of the published data, constitute a heterogeneous mixture of mono- and di-RLs. Thus, it is interesting to evaluate the individual biological properties of purified mono- and di-RLs.

*Pseudomonas aeruginosa* KT1115 was verified as a high di-RLs-yielding strain, with di-RLs comprising a major proportion of the final product (mono-RLs: di-RLs = 11.47: 88.53). The reported rhamnolipids with different mono-/di-RLs ratios have been summarized in Table 2. Many studies have shown that the di-rhamnolipid proportion varied greatly, and was mostly between 23 and 79% in RLs product from various *P. aeruginosa* strains (Matasandoval et al., 1999; Jadhav et al., 2018). The *rhlC* genes, which encode the rhamnosyltransferases involved in di-rhamnolipid production in *P. aeruginosa*, may affect the distribution of mono- and di-RLs as well as different carbon sources (Nicolò et al., 2017). Thus, the mechanism of high di-rhamnolipid production in *P. aeruginosa* KT1115 merits further study.

An effective RLs purification method was established in this study and we introduced a petroleum ether purification step in the purification procedure for the first time. This is a simple and effective purification method with a RLs recovery rate as high as 91.82%, affording RLs with a good purity of 89%. Membrane separation is a good choice for continuous separation of rhamnolipids. According to the literature, membrane separation enables more than 90% rhamnolipid recovery (Isa et al., 2008; Witek-Krowiak et al., 2011). However, the method used in this study achieves efficient separation of rhamnolipids without expensive equipment foundations and cumbersome operations compared with membrane separation. Subsequently, silica gel column chromatography was used to separate mono- and di-RLs with a suitable elution protocol, and it can provide guidance for future research. However, it is worth noting that the specific purification process will be different for varying mono/di ratios. For example, Yuan et al. used chloroform: methanol at ratios of 10:1, 2:1, 1:1, and 1:2 (v/v) as mobile phase to separate mono-/di-RLs from a mixture with di-RLs constituting over 70% of the total (Yuan et al., 2007).

Based on the newly established purification and separation protocol, purified RLs, and mono-/di-RLs were obtained, and the properties of the purified mono- and di-RLs were systematically evaluated. At present, the performance of di-RLs has not been studied after mono- and di-RLs separation, and their performance was only inferred from the different ratio of the di-RLs (Wen-Jie et al., 2012; Feng et al., 2018). In this study, we systematically evaluated properties of the purified mono- and di-RLs, and it shows that di-RLs have much lower CMCs than mono-RLs and higher emulsifying activity than other chemical surfactants including to of SDS and Tween 80. Moreover, di-RLs showed better performance on the characters of temperatures, pH values, and ionic strengths, indicated that they can be used in extreme environments (Banat et al., 2000).

## Conclusions

Compared to mono-RLs, di-RLs possesses higher surface activity, which show great potential in surfactant industry. In this study, the newly isolated *P. aeruginosa* KT1115 produced 44.39 g/L of RLs in a 5 L bioreactor after process optimization. Efficiently 4-step RLs recovery method was established and 91.82% RLs recovery rate with purity of 89% was obtained. The newly established separation process provided a favorable foundation for evaluation of their properties. Afterwards, the results of ESI-MS revealed that *P. aeruginosa* KT1115 is a high di-RLs-yielding strain producing a high di-RLs proportion (mono-RLs: di-RLs = 11.47: 88.53). At the same time, the properties of purified mono- and di-RLs were assessed by TLC, CMC, and EI_24_ analyses. This study provides the basic knowledge on high di-rhamnolipid-yielding *P. aeruginosa* KT1115 and surfactant characteristics of mono/di-RLs. Further genome and transcriptome analysis of the genes involved RLs production may help us to understand the molecular mechanism and genetics of high di-RLs production of strain KT1115.

## Data Availability Statement

All datasets generated for this study are included in the manuscript/supplementary files.

## Author Contributions

JZ, RX, and SL designed and did all experiments, analyzed data, and wrote the paper. NX, FX, WZ, MJ, and WD contributed to the conception of the experiments and supervise the overall project.

### Conflict of Interest

The authors declare that the research was conducted in the absence of any commercial or financial relationships that could be construed as a potential conflict of interest.
